# Supplementary figures and images for: Marine Natural Products and Drug Resistance in Latent Tuberculosis
Source: Mar Drugs. 2019 Sep 26;17(10):549. doi: 10.3390/md17100549 (PMC6836121; doi:10.3390/md17100549)

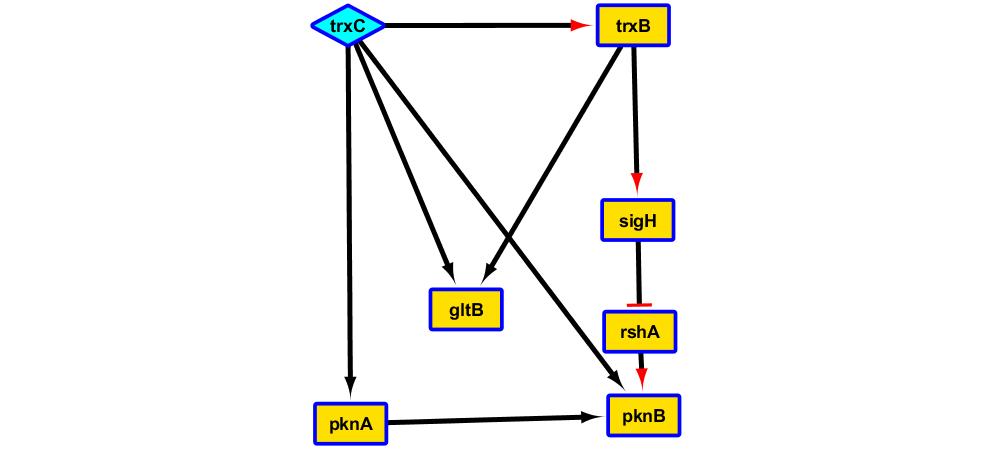


**Figure S2. Longest Path generated through cystoscope plugin, Path linker.**

Supplement: Supplementary file 1 [file marinedrugs-17-00549-s001.zip › All Suplimentary Files/S1.docx]

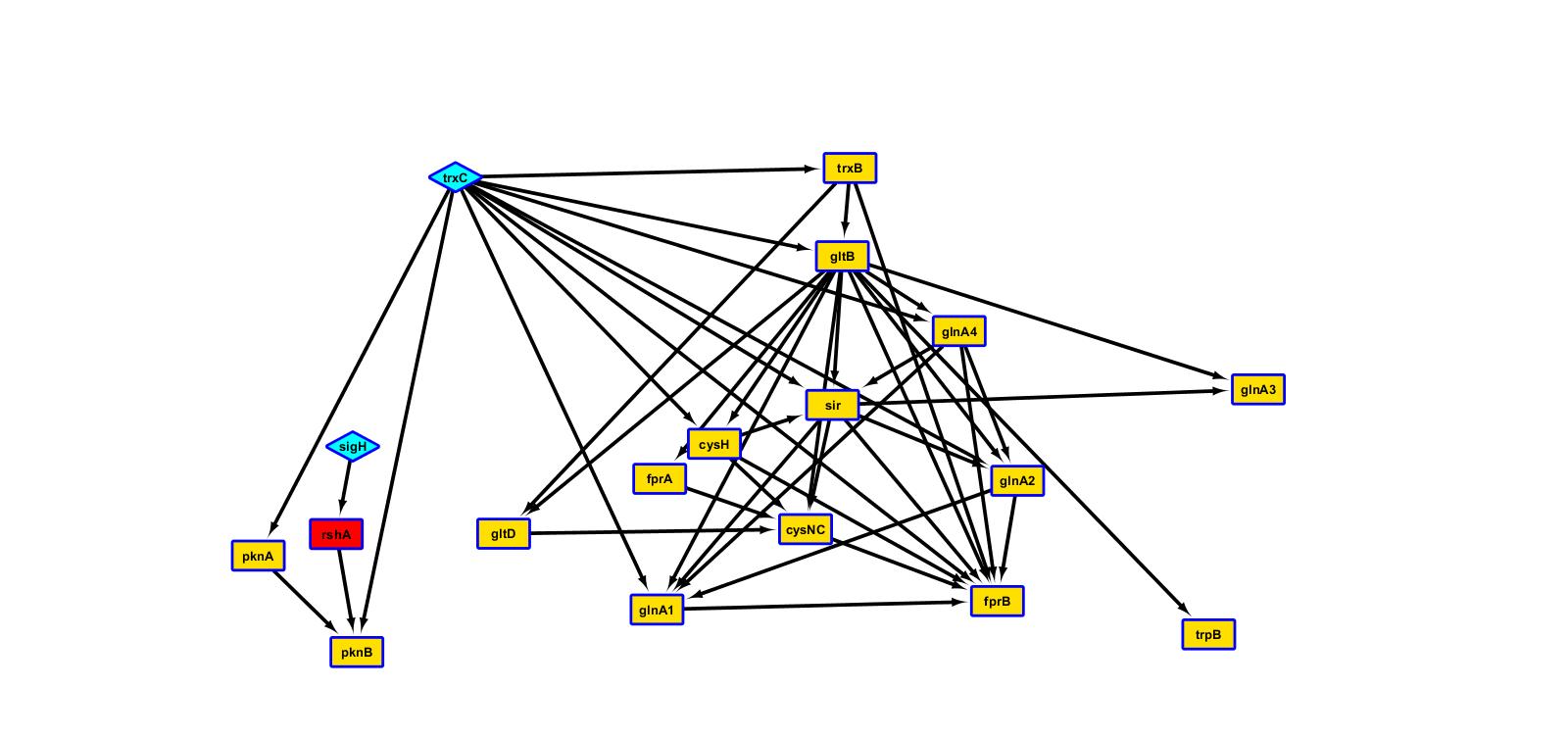


Pathlinker identified paths in network file of string.

Supplement: Supplementary file 1 [file marinedrugs-17-00549-s001.zip › All Suplimentary Files/S2-pathlinker path.docx]

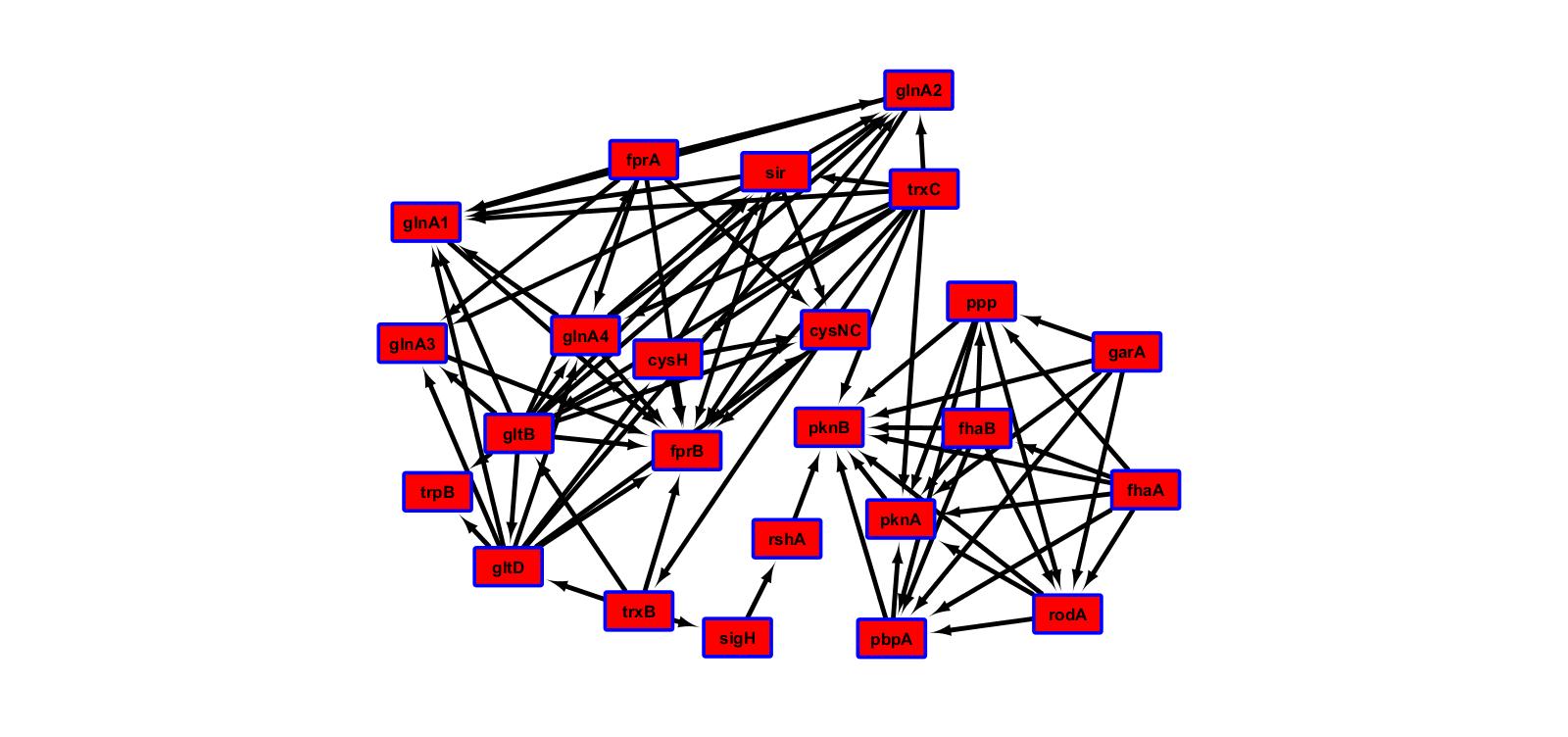


Figure S5. PknB and SigH network generated in string.

Supplement: Supplementary file 1 [file marinedrugs-17-00549-s001.zip › All Suplimentary Files/S3-PknB-SigH.docx]

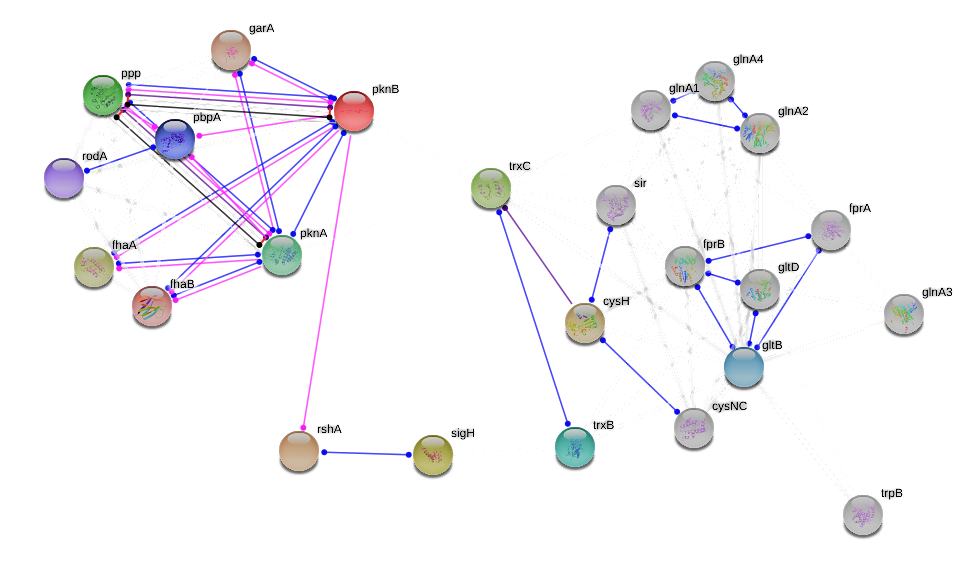


Figure S6. PknB, SigH, and RshA containing network in string database.

Supplement: Supplementary file 1 [file marinedrugs-17-00549-s001.zip › All Suplimentary Files/S4-PknB-SigH-RshA.docx]
